# Concurrent acute illness and comorbid conditions poorly predict antibiotic use in upper respiratory tract infections: a cross-sectional analysis

**DOI:** 10.1186/1471-2334-7-47

**Published:** 2007-05-30

**Authors:** Ilene H Zuckerman, Eli N Perencevich, Anthony D Harris

**Affiliations:** 1 Department of Pharmaceutical Health Services Research, School of Pharmacy, University of Maryland, Baltimore, 220 Arch Street, Baltimore, Maryland, 21201, USA; 2 VA Maryland Health Care System, Baltimore, Maryland, 21201, USA; 3 Department of Epidemiology and Preventive Medicine, School of Medicine, University of Maryland, Baltimore, 100 North Greene Street Lower Level, Baltimore, Maryland, 21201, USA

## Abstract

**Background:**

Inappropriate antibiotic use promotes resistance. Antibiotics are generally not indicated for upper respiratory infections (URIs). Our objectives were to describe patterns of URI treatment and to identify patient and provider factors associated with antibiotic use for URIs.

**Methods:**

This study was a cross-sectional analysis of medical and pharmacy claims data from the Pennsylvania Medicaid fee-for-service program database. We identified Pennsylvania Medicaid recipients with a URI office visit over a one-year period. Our outcome variable was antibiotic use within seven days after the URI visit. Study variables included URI type and presence of concurrent acute illnesses and chronic conditions. We considered the associations of each study variable with antibiotic use in a logistic regression model, stratifying by age group and adjusting for confounders.

**Results:**

Among 69,936 recipients with URI, 35,786 (51.2%) received an antibiotic. In all age groups, acute sinusitis, chronic sinusitis, otitis, URI type and season were associated with antibiotic use. Except for the oldest group, physician specialty and streptococcal pharyngitis were associated with antibiotic use. History of chronic conditions was not associated with antibiotic use in any age group. In all age groups, concurrent acute illnesses and history of chronic conditions had only had fair to poor ability to distinguish patients who received an antibiotic from patients who did not.

**Conclusion:**

Antibiotic prevalence for URIs was high, indicating that potentially inappropriate antibiotic utilization is occurring. Our data suggest that demographic and clinical factors are associated with antibiotic use, but additional reasons remain unexplained. Insight regarding reasons for antibiotic prescribing is needed to develop interventions to address the growing problem of antibiotic resistance.

## Background

Upper respiratory tract infections (URIs) are one of the most frequent reasons patients see their physicians. [1] Antibiotics are generally not indicated for the treatment of URIs as the etiology is usually viral and antibiotics have no demonstrated benefit [1-5]. Recognition of this problem has resulted in numerous efforts to reduce the use of antibiotics in patients with URI, in large part to stem the recent increases in the prevalence of antibiotic resistant organisms[4,6,7]. Inappropriate use of antibiotics also leads to an unnecessarily high incidence of adverse drug reactions and additional costs with few benefits. Despite this knowledge, physicians continue to prescribe antibiotics in more than 45% of URI episodes [8-10].

Although other studies have explored factors associated with antibiotic use for URIs, few studies have examined the importance of comorbidity [11-14]. In fact, little information is available regarding the importance of comorbidity using administrative claims databases. The objectives of this study were to describe patterns of upper respiratory tract infection treatment and to identify patient factors and provider specialties associated with antibiotic use. In particular, since some comorbid conditions and comorbid acute illnesses may justify the use of antibiotics for URI episodes, we were interested in the predictive power of comorbid chronic conditions and comorbid acute illnesses for antibiotic use in URIs.

## Methods

This study was a cross-sectional analysis of medical and pharmacy claims data from the Pennsylvania Medicaid fee-for-service program database. The study population comprised patients in the Pennsylvania Medicaid fee-for-service program (i.e., those not enrolled in a Medicaid health maintenance organization) with a diagnosis of URI. Patients were selected based on medical claims from June 1, 1999 through May 31, 2000, with an office visit coded with one of the following *International Classification of Diseases*,*Ninth Revision, Clinical Modification *[15] diagnostic codes indicating a respiratory tract infection: acute nasopharyngitis (460); acute pharyngitis, excluding streptococcal pharyngitis (462); acute URI of multiple or unspecified sites (465); acute laryngopharyngitis (465.0); acute URI, multiple sites (465.8); acute URI, unspecified site (465.9); acute bronchitis (466.0); influenza with other respiratory manifestations (487.1) and bronchitis not otherwise specified (490).

Since the unit of analysis was the patient and to avoid duplication of patients in the analysis, we randomly selected one URI episode for each patient during the study period to be designated the index URI episode. To be included in the study, patients must have been eligible for Medicaid for at least seven days before and after the service date of the index URI episode, to allow for the opportunity to observe antibiotic use and other acute conditions temporally related to the URI episode.

Patient demographic information was linked to medical and pharmacy claims files. Antibiotic prescription information within seven days after the index URI episode was extracted. The outcome variable was defined as evidence of antibiotic use (yes/no) on the same day as, or within seven days after the index URI episode date. The study variables of interest were (1) presence of concurrent acute illnesses (eight different variables indicating a diagnosis of otitis media, streptococcal pharyngitis, pneumonia, chronic sinusitis, acute sinusitis, urinary tract infection, cellulitis or bacteremia, within seven days before or seven days after the index URI episode); and (2) history of comorbid chronic conditions (defined as presence of one or more claims with a diagnosis of chronic bronchitis, immunodeficiency including HIV/AIDS, solid organ transplant or malignancy within one year prior to the index URI episode) that might impact a physician's decision to prescribe an antibiotic. In addition, analysis included evaluation of potential confounding factors associated with antibiotic use, including the patient's age, sex, race, county of residence (defined as one of six county clusters), season of the URI episode (winter, spring, summer or fall) and specialty of the office visit physician associated with the URI episode (defined as one of four primary specialty groups: family medicine/internal medicine/general practitioner; pediatrics; emergency medicine; all other specialties).

Analyses were conducted using SAS^® ^PC version 8.0 (SAS Institute, Inc., Cary, North Carolina). Patient and provider characteristics were categorized and compared using the chi-square test. Logistic regression was used to estimate the association of each study variable with the probability of antibiotic use when adjusted for gender, race, physician specialty and season of the URI episode. Odds ratios (OR) and 95% confidence intervals (CI) are reported. Since age modified the effect between antibiotic use and several of the other independent variables, we report separate regression models for four age groups (0 to 5 years, 6 to 21 years, 22 to 44 years and ≥ 45 years). The C statistic, which estimates the area under the receiver-operating characteristic (ROC) curve, was used to evaluate the ability of the presence of any concurrent acute illness (aggregated into one dichotomous variable indicating the presence or absence of any of the concurrent acute illnesses) or history of chronic comorbid conditions to distinguish patients who received an antibiotic from patients who did not, while controlling for gender, race, physician specialty and season. Separate C statistics were calculated for each age group stratification. The C statistic reports values from 0.5 (indicating no predictive power) to 1.0 (indicating perfect prediction)[16] Values of less than 0.7 generally indicate poor predictive power.

This analysis was completed as part of an ongoing retrospective drug utilization review (DUR) program for the Pennsylvania Department of Public Welfare (DPW). The DUR program work plan was approved by the University of Maryland Institutional Review Board.

## Results

### Study Population

There were 69,936 unique Medicaid recipients with at least one episode of a URI during the identification period (Table 1). There were a total of 110,677 URI episodes; the number of episodes per patient ranged from 1 to 55, with a median and mode of 1 episode per patient. The mean age was 21.5 (SD 20.4) years, and the median age was 16 years. The study population was primarily female (62.0%) and white (83.9%). More than one third of the patients had a URI episode occurring during the winter season.

**Table 1:** Characteristics of Medicaid patients with an upper respiratory tract infection (URI) episode (n = 69,936)

**Characteristic**	**Number (%)**
Age (years)	
0–5	20,213 (28.9%)
6–21	21,843 (31.2%)
22–44	17,125 (24.5%)
45 and older	10,755 (15.4%)
Gender	
Male	26,602 (38.0%)
Female	43,334 (62.0%)
*Race*	
White	58,663 (83.9%)
Nonwhite	11,273 (16.1%)
Number of URI episodes in one year	
1	46,765 (66.9%)
2	13,839 (19.8%)
3 or more	9,332 (13.3%)
Diagnosis of index URI episode	
Acute bronchitis	19,344 (27.7%)
Common cold	1,818 (2.6%)
Influenza with respiratory symptoms	1,682 (2.4%)
Acute URI, other sites or not specified	47,092 (67.3%)
Presence of concurrent acute illnesses	
Any acute condition	10,012 (14%)
Otitis media	5,505 (8)
Acute sinusitis	2,373 (3)
Chronic sinusitis	1,023 (1)
Pneumonia	575 (< 1)
Strep pharyngitis	540 (< 1)
Cellulitis	117 (< 1)
Urinary tract infection	68 (< 1)
Bacteremia	13 (< 1)
History of chronic comorbid conditions*	
Yes	1,763 (2.5%)
No	68,173 (97.5%)
Physician specialty for index URI episode	
Family practice, general practice or internal medicine	28,320 (40.5%)
Pediatrics	4,139 (5.9%)
Emergency medicine	4,411 (6.3%)
Other specialty	33,066 (47.3%)
Season of index URI episode	
Winter	24,575 (35.1%)
Spring	15,786 (22.6%)
Summer	10,650 (15.2%)
Fall	18,925 (27.1%)

* One or more claims with a diagnosis of chronic bronchitis, immunodeficiency including HIV/AIDS, solid organ transplant or malignancy, one year prior to the index URI episode.

### Frequency and Characteristics of Antibiotic Treatment

Among the 69,936 recipients included in the analysis, 35,786 (51.2%) received an antibiotic on the same day as, or within seven days after their index URI episode. Among the patients who received an antibiotic, 27,656 (77.3%) had an antibiotic prescription on the same day as the index URI episode and 34,091 (95.3%) had an antibiotic prescription within three days of the index URI episode. Among those receiving an antibiotic, 33,900 (94.7%) had only one antibiotic prescription; 1,815 (5.1%) had two prescriptions, and 71 (0.2%) had three or four antibiotic prescriptions on the same day or within seven days after the index URI episode. When excluding the 10,012 patients with a concurrent acute illness that may have justified an antibiotic, antibiotic prevalence was 47.8%.

In the bivariate analysis, antibiotic use varied by age group (p < 0.0001), with the lowest prevalence (44.9%) in the youngest age group and the highest prevalence (56.2%) in the oldest age group. The most frequently prescribed antibiotic was amoxicillin (36.9% of antibiotic prescriptions), followed by azithromycin (19.6%), clarithromycin (7.1%), amoxicillin/clavulanate (6.8%) and erythromycin (5.1%).

### Factors Associated with Antibiotic Use

The presence of any acute concurrent illness was significantly associated with antibiotic use, the strength of association was greatest in the youngest age group (OR 4.63, 95% CI 4.29, 5.00). (Table 2). Across all age groups except age > 45 years, physician specialty was significantly associated with antibiotic use; family physicians and emergency room physicians were more likely to be the prescribing physician compared to other specialties and pediatricians (data not shown).

**Table 2:** Adjusted odds ratios (OR) for antibiotic use in upper respiratory tract infections (URI) among Pennsylvania Medicaid fee-for-service recipients, June 1, 1999 through May 31, 2000, by Age Group*

	**Odds Ratios for Antibiotic Use**							
	**Age 0 – 5 years**		**Age 6 – 21 years**		**Age 22 – 44 years**		**Age ≥ 45 years**	

	**OR**	**95% CI**	**OR**	**95% CI**	**OR**	**95% CI**	**OR**	**95% CI**
Any concurrent acute illness	4.63	4.29–5.00	2.83	2.58–3.10	1.84	1.68–2.02	1.73	1.51–2.00
Acute Sinusitis	3.70	2.71–5.04	2.80	2.33–3.36	1.90	1.68–2.17	2.17	1.75–2.68
Bacteremia	2.11	0.50–8.88	--	--	0.21	0.02–1.90	--	--
Cellulitis	1.41	0.63–3.15	1.38	0.61–3.14	2.19	1.11–4.33	0.90	0.44–1.84
Chronic Sinusitis	4.01	2.69–5.97	2.98	2.29–3.88	2.02	1.62–2.51	1.94	1.39–2.73
Otitis media	4.56	4.20–4.95	2.88	2.53–3.28	1.60	1.34–1.91	1.70	1.21–2.41
Pneumonia	1.71	1.27–2.29	1.93	1.28–2.92	1.18	0.84–1.64	0.96	0.68–1.35
Strep pharyngitis	3.12	2.10–4.63	1.61	1.26–2.06	1.39	0.95–2.05	0.76	0.31–1.84
Urinary tract infection	4.31	0.45–41.62	3.02	1.00–9.14	2.95	1.17–7.45	0.73	0.31–1.73
History of chronic conditions^§^	1.47	0.96–2.27	1.06	0.75–1.51	1.10	0.93–1.29	1.11	0.97–1.28
URI Diagnosis								
Influenza	1	Reference	1	reference	1	reference	1	reference
Common cold	1.64	1.20–2.25	1.27	0.96–1.67	1.42	1.07–1.88	1.07	0.75–1.53
Acute bronchitis	4.83	3.63–6.42	5.84	4.75–7.24	4.28	3.52–5.20	3.83	3.07–4.78
Other sites or not specified	1.74	1.32–2.30	2.46	2.00–3.02	2.54	2.09–3.08	2.17	1.73–2.71

CI denotes confidence interval.

--denotes OR cannot be calculated since there were no episodes of bacteremia.

*Adjusted for gender, race, physician specialty and season of URI. The probability of receiving an antibiotic at a visit was the dependent variable.

^§^Chronic conditions include chronic bronchitis, immunodeficiency including HIV/AIDS, solid organ transplant or malignancy.

For each age group, the independent effects of each of the study variables of antibiotic use was analyzed using a multivariate logistic regression model, with antibiotic use (yes/no) as the outcome variable (Table 2). After controlling for gender, race, physician specialty and season in each age group, concurrent diagnoses of acute sinusitis, chronic sinusitis and otitis media were associated with antibiotic use, with the odds ratios for receiving an antibiotic for each of theses acute illnesses highest in the youngest age group.

In children ages 0 to 5 years, a concurrent diagnosis of otitis media (OR 4.56, 95% CI 4.20, 4.95), streptococcal pharyngitis (OR 3.12, 95% CI 2.10, 4.63), chronic sinusitis (OR 4.01, 95% CI 2.93, 6.70), acute sinusitis (OR 3.85, 95% CI 2.69, 5.97) and pneumonia (OR 1.71, 95% CI 1.27, 2.29) increased the likelihood of receiving an antibiotic.

Patients aged 6 to 21 years were more likely to receive an antibiotic for a URI if they had a concurrent diagnosis of otitis media (OR 2.88, 95% CI 2.53, 3.28), chronic sinusitis (OR 2.98, 95% CI 2.29, 3.88), acute sinusitis (OR 2.80, 95% CI 2.33, 3.36) streptococcal pharyngitis (OR 1.61, 95% CI 1.26, 2.06) and pneumonia (OR 1.93, 95% CI 1.28, 2.92). Additionally, this age group was more likely to receive an antibiotic if there was a concurrent urinary tract infection (OR 3.02, 95% CI 1.00, 9.14). In the 22 to 44 year old age group, four of the eight concurrent acute respiratory illnesses were significantly associated with antibiotic use for a URI [concurrent acute sinusitis (OR 1.90, 95% CI 1.68–2.17), chronic sinusitis (OR 2.02, 95% CI 1.62–2.51), cellulitis (OR 2.19, 95% CI 1.11–4.33), otitis media (OR 1.60, 95% CI 1.34–1.91)]. Concurrent urinary tract infection (OR 2.95, 95% CI 1.17–7.45) was also associated with antibiotic use. Concurrent acute illnesses in the oldest group (age ≥ 45 years) that were associated with antibiotic use included acute sinusitis (OR 2.17, 95% CI 1.75–2.68), chronic sinusitis (OR 1.94, 95% CI 1.39–2.73), acute sinusitis (OR 2.17, 95% CI 1.75–2.68) and otitis media (OR 1.70, 95% CI 1.21–2.41). A history of chronic comorbid conditions (i.e., one or more claims with a diagnosis of chronic bronchitis, immunodeficiency including HIV/AIDS, solid organ transplant or malignancy, one year prior to the index URI episode) was not associated with antibiotic use in any of the age groups.

The C statistic (area under the ROC curve) was used to assess the ability of the presence of the regression models to discriminate patients who received antibiotics from those who did not. We report the C-statistics for the 'any acute condition' models (models corresponding to the first row of results in Table 2) and for the 'history of chronic conditions' models (models corresponding to the tenth row of results in Table 2). C statistics ranged from 0.54 (history of chronic comorbid conditions in the 22 to 44 year age group) to 0.67 (presence of concurrent acute illness in the 0 to 5 year age group), suggesting that these models have poor to fair predictive value.

## Discussion

In the Pennsylvania Medicaid fee-for-service population during the study period, over one-half of recipients with an upper respiratory tract infection received an antibiotic on the same day as or within seven days after a physician visit for a URI. Even among those patients without a concurrent acute illness that may justify an antibiotic, antibiotic use was high (47.8%). These findings are consistent with other similar analyses, which found 44% to 60% prevalence of antibiotic prescribing for colds or URIs[8,9,17,18]. The high prevalence indicates that potentially inappropriate antibiotic use is high and should be a target for antibiotic education and management to reduce excess use.

Antibiotic resistance is a worldwide public health issue, resulting in emerging antimicrobial resistance and costing $1.3 billion in the United States in 1992[19]. Unfortunately, the extent of the problem is not fully realized since surveillance of drug-resistance is limited[20]. Evidence for a causal association between antimicrobial use and resistance is supported by ecological data (changes in use parallel changes in prevalence of resistance). Also, there is evidence of increased prevalence of resistance in nosocomial strains compared to community-acquired strains, association of prior antibiotic use with nosocomial outbreaks and association of increased antibiotic use with increased resistance rates[21,22].

To address prescribing factors, educational and administrative interventions have been proposed [23-31]. Administrative interventions are generally restrictive in nature and include prior authorization, formulary restriction, ordering forms and cycling. Educational interventions may include dissemination of susceptibility information, use of computer-based algorithms, and academic detailing. An understanding of the factors contributing to potentially inappropriate antibiotic use can help guide policy makers to design an effective educational or administrative intervention.

Our findings suggest that the presence of certain concurrent acute illnesses increase the likelihood of antibiotic use for a URI. It is possible those antibiotics were actually prescribed for these other acute conditions and that the URI was a coincidental diagnosis. Hence, it could be argued that for some patients the prescription of antibiotics may have been indicated.

However, antibiotic use was highly prevalent (47.8%) even among those without a coincidental diagnosis. In the final regression models coincidental diagnoses only had fair to poor ability to distinguish patients who received an antibiotic from patients who did not, as indicated by the C statistics. Although the presence of illnesses such as otitis media or streptococcal pharyngitis was significantly associated with antibiotic use, they did not help to discriminate patients who received an antibiotic during a URI from those who did not. Thus, the reasons why physicians prescribe antibiotics for URIs is complex and is not fully explained by regression models of this study. Several reasons have been cited for inappropriate prescribing of antibiotics. These include patient expectation, [32-35] physicians' perception of patients' expectations,[32,35-37] patients' lack of education,[35,38] and economic pressure[35,38]. Our findings suggest that more research is needed in understanding why antibiotics are being used for URIs in adults, as well as in children.

This paper explored predictive factors of antibiotic use for URI in an administrative database namely a Medicaid population. The concurrent presence of an acute illness such as cellulitis or UTI may explain the observed antibiotic prescription that was contemporaneous to a URI episode. In addition, we chose to explore the importance of comorbidity in predicting antibiotic use. For some chronic comorbid conditions, such as chronic malignancy or immunodeficiency, antibiotic use may be medically indicated for URIs. Thus, we chose a limited number of comorbid conditions, that we believe justified antibiotic use. Other studies have used a wider range of comorbid conditions that included diabetes, cardiovascular disease. These additional comorbid conditions may be important risk factors in that they increase antibiotic prescribing. However, we chose not to include these additional conditions because their presence generally does not justify antibiotic use.

One limitation of this study was that validation of the medical and prescription claims data against the primary data in the actual medical record was not possible due to limited resources. However, Medicaid prescription claims tend to be a reliable source of drug availability[39].

Reimbursement is linked to accurate billing, though the reliability of the diagnosis on the medical service claim is less certain since diagnosis generally does not influence reimbursement directly. This may result in misclassification of URI-related events due to incorrect data entry or wrong code assignment. For example, we included ICD-9-CM code 490 (bronchitis not otherwise specified) in our definition of URI; this code may also include patients with COPD or acute exacerbation of chronic bronchitis. In addition, the severity of the URI or the actual indication for the antibiotic prescribed cannot be definitively determined from claims data. However, we did attempt to link the antibiotic to the URI episode temporally and control for other potential antibiotic indications in the analysis. Another limitation is that there may be misclassification of chronic disease history. Although we searched the administrative claims for evidence of chronic conditions for one year before the index URI episode, only 64% of subjects were eligible for an entire year. However, when we reran the 'history of chronic conditions' models for only those subjects with one year of eligibility prior to the URI episode, we obtained results to those in Table 2 (row 10), and nearly identical C statistics to the reported models.

Finally, these findings may not be generalizable to populations other than Medicaid, since Medicaid patients are differentiated by low economic status, source of care and other factors that may affect drug utilization.

## Conclusion

This analysis suggests that demographic and clinical factors are associated with the prescription of an antibiotic for a URI, but much of the antibiotic use remains unexplained by acute illnesses and comorbid conditions. More insight into the reasons for antibiotic prescribing needs to be gained, which will lead to novel interventions that may hopefully curb the growing problem of antibiotic resistance.

## Competing interests

The author(s) declare that they have no competing interests.

## Authors' contributions

IZ, EP and AH have made substantial contributions to conception and design, data analysis and interpretation of data, have been involved in drafting the manuscript or revising it critically for important intellectual content and have given final approval of the version to be published

## Pre-publication history

The pre-publication history for this paper can be accessed here:


